# Functional characterization of a *Penicillium chrysogenum* mutanase gene induced upon co-cultivation with *Bacillus subtilis*

**DOI:** 10.1186/1471-2180-14-114

**Published:** 2014-05-06

**Authors:** Ishwar Bajaj, Tânia Veiga, Dino van Dissel, Jack T Pronk, Jean-Marc Daran

**Affiliations:** 1 Department of Biotechnology, Delft University of Technology, Julianalaan 67, 2628 BC Delft, the Netherlands; 2 Kluyver Centre for Genomics of Industrial Fermentation, Julianalaan 67, 2628 BC Delft, the Netherlands; 3 Platform for Green Synthetic Biology, P.O. Box 5057, 2600 GA Delft, the Netherlands

**Keywords:** Time-course transcriptional analysis, Mixed culture, Chemostat-based transcriptomics, Mutanase, Heterologous expression, Penicillium chrysogenum, Bacillus subtilis

## Abstract

**Background:**

Microbial gene expression is strongly influenced by environmental growth conditions. Comparison of gene expression under different conditions is frequently used for functional analysis and to unravel regulatory networks, however, gene expression responses to co-cultivation with other microorganisms, a common occurrence in nature, is rarely studied under laboratory conditions. To explore cellular responses of the antibiotic-producing fungus *Penicillium chrysogenum* to prokaryotes, the present study investigates its transcriptional responses during co-cultivation with *Bacillus subtilis*.

**Results:**

Steady-state glucose-limited chemostats of *P. chrysogenum* grown under penillicin-non-producing conditions were inoculated with *B. subtilis*. Physiological and transcriptional responses of *P. chrysogenum* in the resulting mixed culture were monitored over 72 h. Under these conditions, *B. subtilis* outcompeted *P. chrysogenum*, as reflected by a three-fold increase of the *B. subtilis* population size and a two-fold reduction of the *P. chrysogenum* biomass concentration. Genes involved in the penicillin pathway and in synthesis of the penicillin precursors and side-chain were unresponsive to the presence of *B. subtilis*. Moreover, *Penicillium* polyketide synthase and nonribosomal peptide synthase genes were either not expressed or down-regulated. Among the highly responsive genes, two putative α-1,3 endoglucanase (mutanase) genes *viz* Pc12g07500 and Pc12g13330 were upregulated by more than 15-fold and 8-fold, respectively. Measurement of enzyme activity in the supernatant of mixed culture confirmed that the co-cultivation with *B. subtilis* induced mutanase production. Mutanase activity was neither observed in pure cultures of *P. chrysogenum* or *B. subtilis*, nor during exposure of *P. chrysogenum* to *B. subtilis* culture supernatants or heat-inactivated *B. subtilis* cells. However, mutanase production was observed in cultures of *P. chrysogenum* exposed to filter-sterilized supernatants of mixed cultures of *P. chrysogenum* and *B. subtilis*. Heterologous expression of Pc12g07500 and Pc12g13330 genes in *Saccharomyces cerevisiae* confirmed that Pc12g07500 encoded an active α-1,3 endoglucanase.

**Conclusion:**

Time-course transcriptional profiling of *P. chrysogenum* revealed differentially expressed genes during co-cultivation with *B. subtilis*. Penicillin production was not induced under these conditions. However, induction of a newly characterized *P. chrysogenum* gene encoding α-1,3 endoglucanase may enhance the efficacy of fungal antibiotics by degrading bacterial exopolysaccharides.

## Background

*Penicillium chrysogenum* is widely used for industrial production of β-lactam antibiotics such as penicillins and cephalosporins. Current production strains are the result of classical strain development programmes which, over a period spanning more than six decades, has improved productivity by several orders of magnitude [1]. Throughout strain improvement, industrial and academic research has sought to understand the molecular basis and physiology of penicillin production by *P. chrysogenum* based on research in pure cultures.

The complete genome sequence of *P. chrysogenum*[2] revealed 20 putative polyketide synthase genes (PKS), 10 non-ribosomal peptide synthase (NRPS) genes and 2 genes encoding hybrid NRPS-PKS enzymes [3,4]. Most of these genes are not significantly transcribed under standard laboratory conditions [5-10] and, consequently, their functional and physiological roles remains to be determined.

The contribution of many secondary metabolites to evolutionary fitness of the producing organism remains unknown and subject to debate [11-14]. It is generally assumed that fungal penicillin biosynthesis has primarily evolved as a mechanism to combat competing bacteria. However, there is no evidence that fungal penicillin biosynthesis is triggered by the presence of bacteria. Moreover, there is no a priori reason to assume that synthesis of fungal antibiotics it is single-layer defense mechanism. For example, NRPS or PKS gene clusters may enable the synthesis of compounds that complement and/or augment the action of penicillins.

Several strategies have been successfully applied to activate and characterize cryptic gene clusters in filamentous fungi [15]. These methods include inactivation of biosynthesis genes [16], heterologous expression [17] and simulation of conditions that might activate gene clusters in natural environments [5,6,18]. A successful example the latter approach was achieved by co-culturing *Aspergillus nidulans* with *Streptomyces hygroscopicus.* Genome wide expression analysis of *A. nidulans* revealed that co-cultivation led to specific expression of a polyketide gene cluster that was silent under standard conditions for laboratory growth of the fungus [18]*.* This example supports the notion that microbial interactions in natural environments may be a direct trigger for production of secondary metabolites and other means of ‘microbial warfare’. Simulation of these interactions by co-cultivation in the laboratory is therefore a rational strategy to identify natural antimicrobial compounds and mechanisms. Indeed, this approach has been successfully used to study interactions between microorganisms [19-22], allowing the identification of a wide range of responses, ranging from increased production of stress-response related compounds [23], induction of biofilm formation and modulation of virulence expression [20]. By providing in-depth knowledge on interaction mechanisms it provides an important tool for understanding the physiology of microorganisms, communication between microbial species and drug discovery [18].

The dynamic nature of microbial interactions in mixed cultures limit the value of static (single time point) gene expression studies. Time-course expression experiments, in which transcriptional responses to an external stimulus are recorded over time [24] have been applied successfully to a wide range of perturbations [25-29].

The original discovery of penicillin by Fleming was unintentionally based on a co-cultivation experiment, involving interaction of a *Penicillium* strain with bacteria on solid medium plates [30]. Surprisingly however, interaction of this fungus with prokaryotes has subsequently not been studied in detail. The objective of the present study is to investigate the response of *P. chrysogenum* to co-cultivation with *B. subtilis*, a penicillin-sensitive Gram-positive bacterium. To this end, steady-state chemostat cultures of *P. chrysogenum* Wisconsin 54–1255 were inoculated with *B. subtilis*. The dynamic transcriptional and physiological responses of *P. chrysogenum* in the resulting mixed cultures were monitored and analyzed. Several differentially expressed genes potentially reflected responses to the bacterium. To test whether any bacterial signaling molecules are responsible for differential expression of selected fungal genes, *P. chrysogenum* cultures were supplemented with supernatant of *B. subtilis* cultures, supernatants from mixed cultures and with heat-inactivated *B. subtilis*. The specific transcriptional responses, identified using microarray, were verified by analysis of fermentation broth and by functional characterization of selected *P. chrysogenum* genes in *S. cerevisiae*.

## Results

### Co-cultivation of *P. chrysogenum and B. subtilis* and physiology

**Figure 1:**
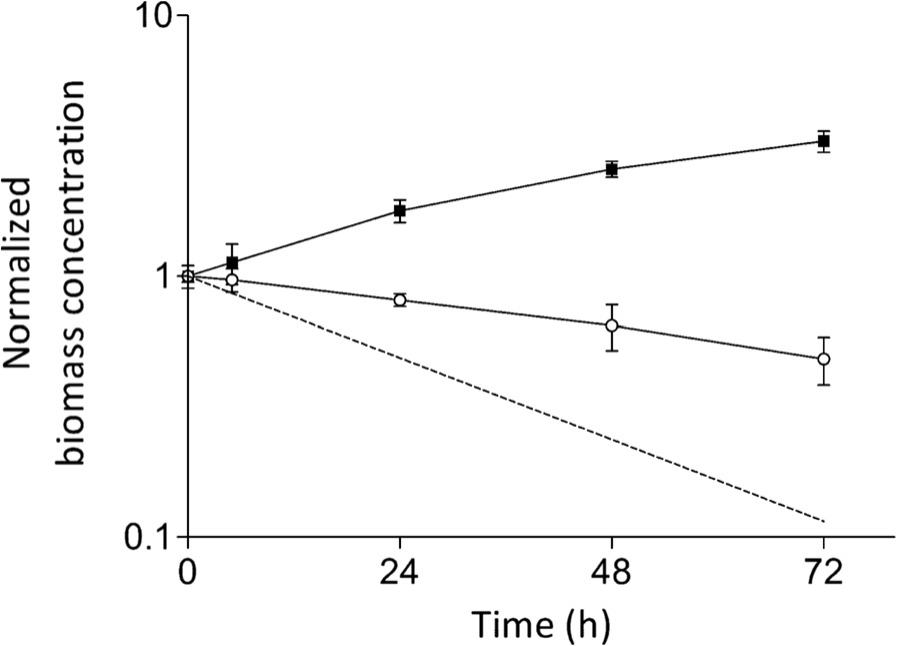
**Dynamics of *P. chrysogenum *and *B. subtilis *during co-cultivation.** At t = 0 h, a steady-state, glucose-limited chemostat culture of *P. chrysogenum*, grown in the absence of phenylacetate and at a dilution rate of 0.03 h^-1^, was inoculated with *B. subtilis* cultures. The data represents the t = 0 h normalized biomass concentration of *P. chrysogenum* (○) and *B. subtilis* (●). The dashed line represents wash-out at zero specific growth rate. Data points represent average and mean deviation of duplicate experiments.

The interaction between *P. chrysogenum* and *B. subtilis* was studied in controlled chemostat experiments. *P. chrysogenum* strain Wisconsin 54–1255, an early strain of the penicillin strain improvement lineage [2,31], was grown in an aerobic, glucose-limited chemostat culture at a dilution rate of 0.03 h^-1^. This dilution rate is much lower than the maximum specific growth rates of *P. chrysogenum* and *B. subtilis* (μ_max_ = 0.14 h^-1^ and 0.47 h^-1^, respectively) in batch cultures on the synthetic medium used for the chemostat experiments. Phenylacetate (PAA), which is needed for the efficient production of penicillin-G production by *P. chrysogenum*, was not included in growth media, to enable a focus on other, as yet unknown, antibacterial mechanisms in this fungus. When the *P. chrysogenum* chemostat cultures had reached stationary phase, they were inoculated with *B. subtilis*. Although both organisms remained present during the entire 72 h of the co-cultivation experiment, the relative bacterial and fungal populations changed over time. During the mixed culture, *B. subtilis* grew faster than *P. chrysogenum* and, as a consequence, the *B. subtilis* biomass concentration increased by more than three fold while the *P. chrysogenum* biomass concentration was reduced by half over a 72 h period (Figure 1). Comparison of *P. chrysogenum* biomass dynamics with a theoretical washout (μ = 0) at a dilution rate of 0.03 h^-1^ confirmed that the fungal biomass was still growing, but slower than the dilution rate, leading to a declining population size in the reactor (Figure 1).

After the bacterial pulse, the characteristics of the fermentation changed. The chemostat was not in steady state anymore and the total biomass increased from 4.037 ± 0.11 g l^-1^ to 4.977 ± 0.29 g l^-1^ after 72 h of mixed culture. The specific oxygen uptake rate and the specific carbon dioxide production rate were decreased from 0.930 ± 0.021 to 0.746 ± 0.047 mmol g^-1^ h^-1^ and from 0.847 ± 0.038 to 0.718 ± 0.052 mmol g^-1^ h^-1^ respectively during 72 h of mixed culture (Table 1).

**Table 1:** **Physiological parameters of co-cultivation of *P. chrysogenum *** **and *B. subtilis***

**Time elapsed since inoculation with *B. subtilis *** **(h)**	**Total biomass concentration**^**a **^**(g l**^**-1**^**)**	**qO**_**2**_^**a,b **^**(mmol g**^**-1**^ **h**^**-1**^**)**	**qCO**_**2**_^**a,b **^**(mmol g**^**-1**^ **h**^**-1**^**)**
0	4.04 ± 0.11	0.93 ± 0.02	0.85 ± 0.04
24	4.32 ± 0.07	0.90 ± 0.01	0.81 ± 0.06
48	4.69 ± 0.17	0.78 ± 0.03	0.75 ± 0.04
72	4.98 ± 0.29	0.75 ± 0.05	0.71 ± 0.05

At t = 0 h, a steady-state, glucose-limited chemostat culture of *P. chrysogenum*, grown in the absence of phenylacetate and at a dilution rate of 0.03 h^-1^, was inoculated with *B. subtilis* cultures. Physiological parameters are presented as average and mean deviation of three independent replicate experiments.

^a^Total biomass is the sum of *P. chrysogenum* and *B. subtilis* biomass. ^b^Specific rates of oxygen consumption and carbon dioxide production (qO_2_ and qCO_2_) were calculated from biomass concentrations and manual analysis of the gas composition and gas-flow.

**Figure 2:**
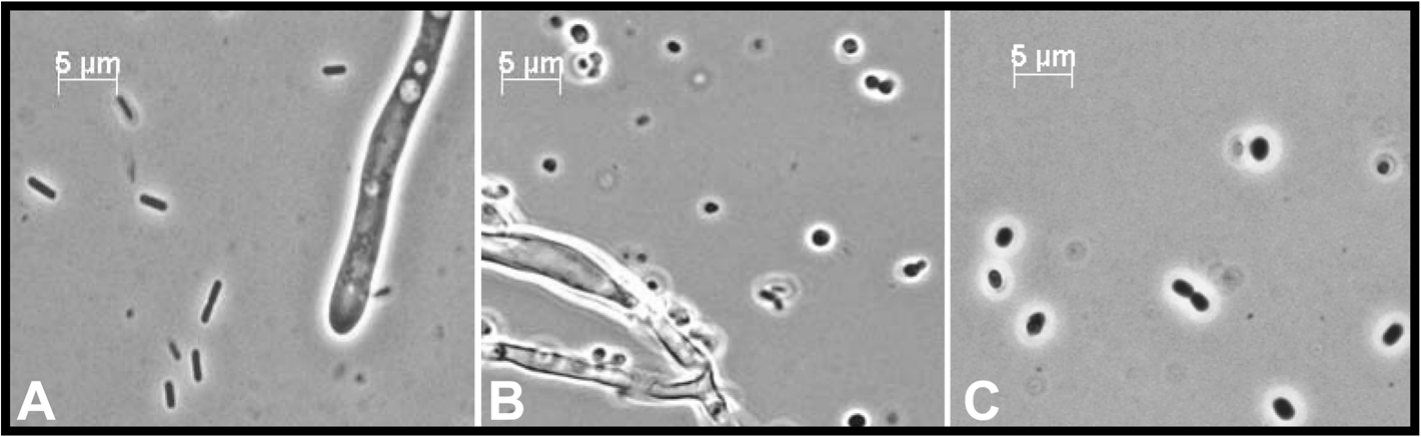
**Change of *B. subtilis *morphology in chemostat cultures. A**: Mixed culture of *B. subtilis* and *P. chrysogenum* at 0 h; *B. subtilis* cells were pregrown in shake-flask culture and addition to a P. chrysogenum glucose-limited chemostat culture. **B**: Mixed culture of *B. subtilis* and *P. chrysogenum* at 72 h. **C**: pure culture of *B. subtilis* after 72 h of glucose-limited cultivation. Pictures taken under oil immersion and 1000X magnification using a Zeiss Axio imager D1 and an Axio Camera.

Microscopic observation revealed no detectable adhesion of bacterial cells to the fungal hyphae and no morphological change in *P. chrysogenum*, but the morphology of *B. subtilis* changed dramatically during co-cultivation, from rod-shaped bacteria to spherical cells (Figure 2). To investigate whether the *B. subtilis* morphology change was caused by the presence of the fungus or the culture conditions, a control glucose-limited chemostat culture experiment under exactly the same conditions (including medium composition and dilution rate) but with a pure culture of *B. subtilis* was performed. As the culture was switched from batch to chemostat mode, the typical rod-shaped morphology *B. subtilis* changed into spherical cells. This indicated that the morphology change during co-cultivation with *P. chrysogenum* was due to the glucose-limited culture conditions rather than to a specific interaction with the fungus.

### Global transcriptional responses of *P. chrysogenum* to co-cultivation with *B. subtilis*

**Figure 3:**
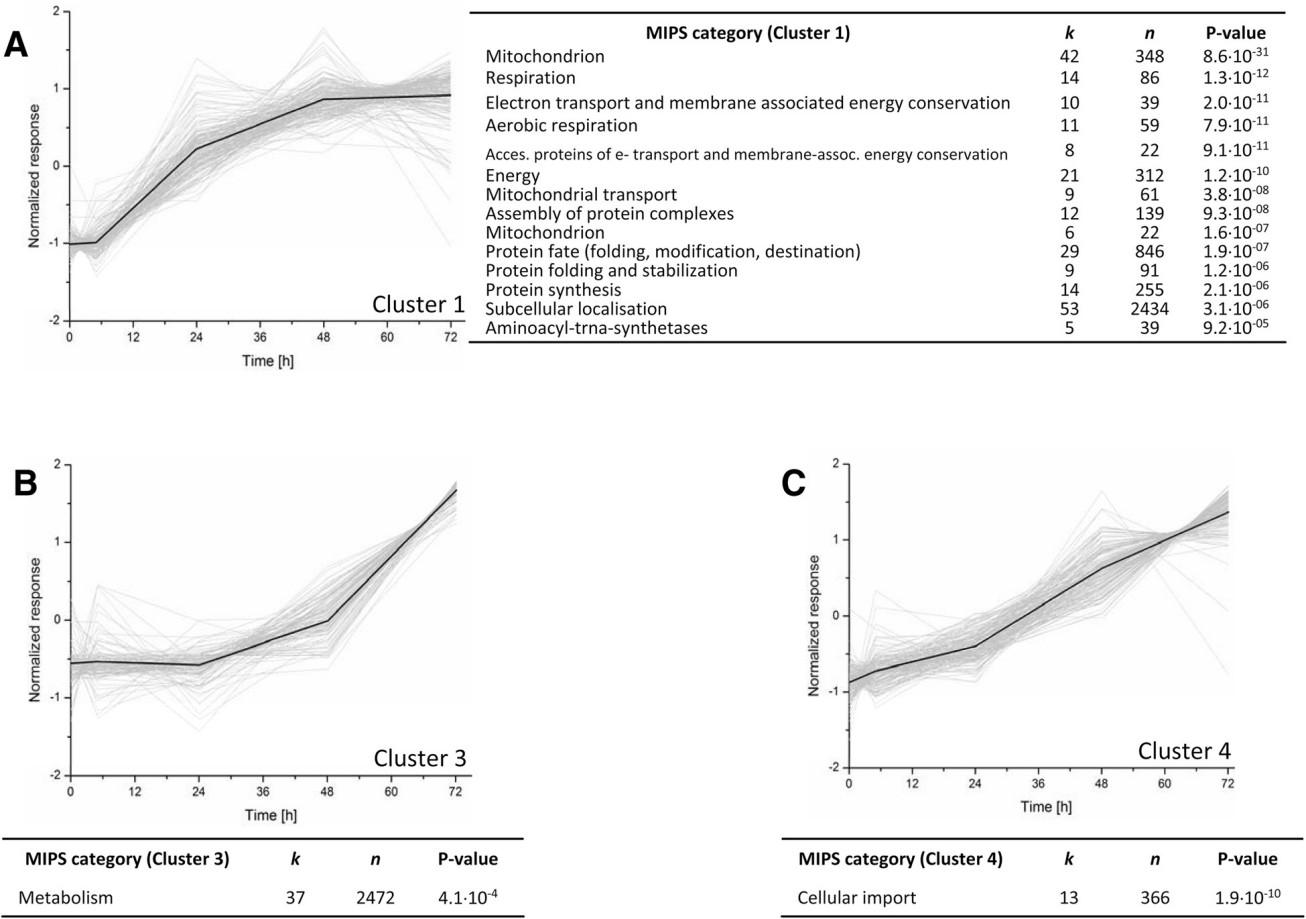
**K-mean clustered gene expression profiles of groups of specific interest (Clusters 1 (A), 3 (B) and 4 (C) over the course of co-cultivation of *P. chrysogenum *with *B. subtilis *and the results of hypergeometric distribution analysis for enrichment of functional categories.** The thick line represents the average of the mean normalized expression data of the genes comprising the cluster. The y-axis represents normalized expression values. Functional categories are mentioned together with their P-value, k representing the number of genes of the corresponding category within the set of differentially expressed genes and n representing the number of genes in the respective functional category in the whole genome.

Dynamic experiments are, inherently, richer in information than steady-state cultivation experiments but also more susceptible to experimental variation. Tight control of experimental conditions and of data quality is therefore of paramount importance [32]. In view of their reproducibility, steady-state chemostat cultures are excellent experimental platforms for dynamic perturbation studies [27,28]. Hence, to identify fungal genes whose expression would change in reaction to co-cultivation with *B. subtilis*, steady-state chemostat cultures of *P. chrysogenum* were inoculated with *B. subtilis* and followed over time (0 h, 5 h, 24 h, 48 h, 72 h). The variation among the same time points in independent duplicate chemostat experiments did not exceed 27%. Analysis with the Significance Analysis of Microarray (SAM) algorithm (http://statweb.stanford.edu/~tibs/SAM/) [33] revealed no fewer than 732 *P. chrysogenum* genes whose expression changed during co-cultivation with *B. subtilis*. K-means clustering of these responsive genes yielded six clusters, with 433 genes displaying an ascending expression profile (clusters 1, 3 and 4) and 299 genes exhibiting a descending profile (cluster 2, 5, 6) during co-cultivation (Figure 3 and Additional file 1: Table S1). Enrichment analyses using Fischer’s Exact statistical test did not reveal any enrichment of MIPS functional categories in clusters with descending profiles (clusters 2, 5 and 6). In contrast, several functional categories were found to be overrepresented in all three clusters comprising ascending profiles (clusters 1, 3 and 4) (Figure 3).

Cluster 1 comprises fast-responding genes, which reached 80% of their maximum expression level within the first 24 h. This cluster showed overrepresentation of genes in the MIPS categories involved in primary metabolism, protein synthesis, folding and stabilization, energy conservation, electron transport, respiration and mitochondrial activity (Figure 3). For example, the gene with the highest fold change after 72 h of co-cultivation (+50-fold), Pc12g10440, showed strong similarity to the alternative oxidase *aox1* from *Aspergillus niger.* Cluster 3 contains genes that exhibited slower response profiles and showed an overrepresentation of genes involved in metabolism (Figure 3). In addition to genes with strong similarity to transporters (permeases, maltose transport protein and high-affinity glucose transporter), this category comprised genes with sequence homology to genes encoding carbohydrate cleaving enzymes such as mutanase, mannosidase and alpha-amylase, to a gene encoding a starvation sensing protein (Pc12g13310) and to a gene showing similarity to polyketide synthase PKS17 from *Botryotinia fuckeliana* (Pc12g13170). Finally, Cluster 4, which comprises genes exhibiting a continuously increasing profile over the 72 h co-cultivation period, showed an overrepresentation of genes involved in cellular import (Figure 3). No fewer than five genes with similarity to the *Kluyveromyces lactis* high-affinity glucose transporter gene *HGT1*. (Pc18g00150; Pc21g19660; Pc22g09740 and Pc21g19770) and the glucose transporter *rco-3* from *Neurospora crassa* (Pc12g02080) were found in this cluster (Additional file 1: Table S1).

### Transcriptional responses of *P. chrysogenum* genes involved in secondary metabolite production to co-cultivation with *B. subtilis*

**Figure 4:**
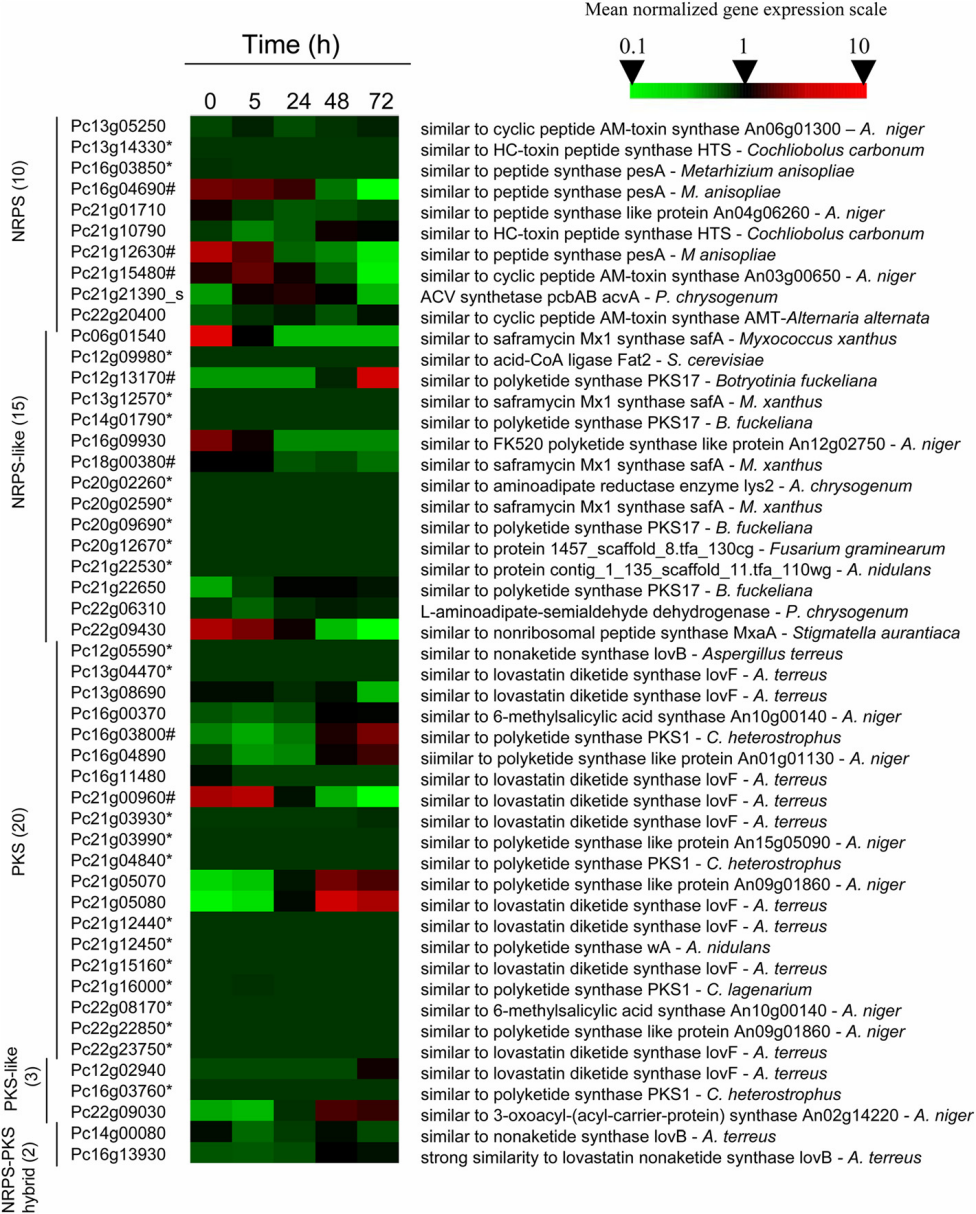
**Expression of *P. chrysogenum *secondary metabolism genes during co-cultivation with *B. subtilis*.** Heat map of transcript levels of genes encoding NRPS (and -like), PKS (and -like) and hybrid NRPS-PKS in strain Wisconsin 54–1255, grown in aerobic glucose-limited chemostat cultures, 0 h, 5 h, 24 h, 48 h and 72 h after addition of *B. subtilis.* The expression data were derived from hybridization intensity of Affymetrix DNA microarrays (DSM PENa520255F) (n = 3). The transcript data were Z-normalized for each experiment. Genes that were not expressed in the mixed culture are marked with *and the seven genes whose transcript profile was deemed significantly different from the null situation are marked with #. The numbers between brackets indicate the numbers of NRPS, NRP-like, PKS, PKS-like and hybrids catalogued in the *P. chrysogenum* genome.

*P. chrysogenum* is primarily known for its ability to synthesize β-lactam antibiotics. Closer inspection of the non-ribosomal peptide penicillin gene cluster (*pcbAB, pcbC* and *penDE*) [34,35] and genes involved in side-chain precursor metabolism did not show differential expression during co-cultivation with *B. subtilis*[36,37]. In addition to the penicillin biosynthesis gene cluster, the *P. chrysogenum* genome sequence [2] harbors 9 other NRPS and 15 NRPS-like, 20 PKS and 3 PKS-like, and 2 hybrid NRPS-PKS gene clusters (Figure 4). All these genes potentially encode NRPS and PKS proteins enabling the synthesis of complex secondary metabolites [3,4,38,39] (Figure 4). Co-cultivation with *B. subtilis* had only small effects on expression of these NRP and PK gene clusters. Only seven individual genes in these clusters were differentially expressed, of which only Pc12g13170 and Pc16g03800, which share sequence similarity with the polyketide synthase PKS17 from *B. fuckeliana*[40] and with the polyketide synthase PKS1 from *Cochliobolus heterostrophus*[41], respectively, were upregulated. However, their absolute expression levels remained very low (Figure 4 and Additional file 1: Table S1).

### *B. subtilis* induced mutanase production in *P. chrysogenum*

**Figure 5:**
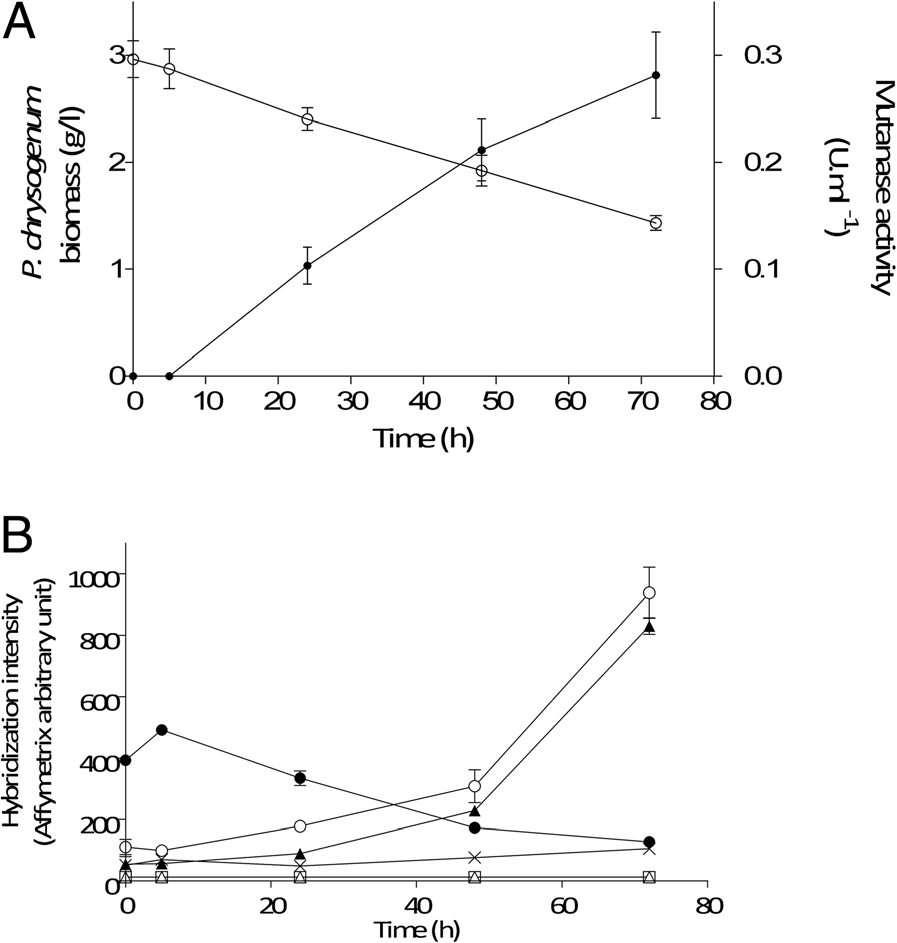
**Mutanase production by *P. chrysogenum *during co-cultivation with *B. subtilis*. A**- fungal biomass (○) and mutanase activity (●) after 0 h, 5 h, 24 h, 48 h and 72 h of co-cultivation with *B. subtilis* in aerobic, glucose-limited chemostat cultures. **B**- Expression of the six putative mutanase genes found in the genome of *P. chrysogenum* during co-cultivation. (●) Pc18g06380, (○) Pc12g13330, (×) Pc13g0113, (∆) Pc15g0400 (▲) Pc12g07500, (□) Pc13g12810. Data are presented as mean and standard deviation of three independent replicates.

Our data indicated that, in the absence of a penicillin side-chain precursor, other non-ribosomal peptides or polyketides did not replace penicillin as an antibiotic against *B. subtilis* in mixed cultures. Among the top 10 of overexpressed *P. chrysogenum* genes, we focused our attention on two genes found in cluster 3. These two genes, Pc12g07500 and Pc12g13330, shared strong sequence similarity to the alpha-1,3-glucanase *mutA* from *Talaromyces. purpurogerus*[42] and were upregulated by 15 fold and 8 fold, respectively after 72 h of co-cultivation with *B. subtilis*. Occurrence of alpha-1, 3-glucanase (mutanase) in response to the presence of *B. subtilis* represented a specific response of *P. chrysogenum*, as mutanase activity was not observed in pure cultures of either *P. chrysogenum* or *B. subtilis*. The presence of mutanase activity in culture supernatants was first observed after 24 h of mixed cultivation. This activity increased from 0.10 ± 0.021 U ml^-1^ at 24 h to 0.28 ± 0.034 U ml^-1^ at 72 h of co-cultivation even though, during this period, the *P. chrysogenum* biomass concentration decreased from 2.40 ± 0.037 g l^-1^ at 24 h to 1.43 ± 0.080 g l^-1^ at 72 h (Figure 5).

**Figure 6:**
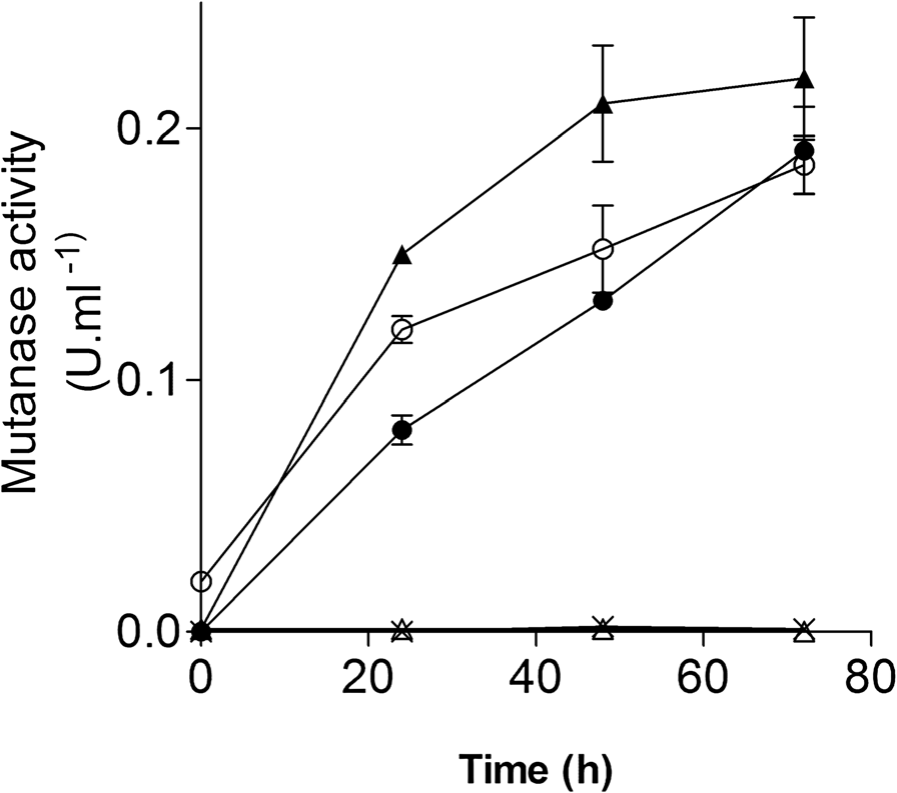
**Mutanase production by *P. chrysogenum *after addition of cell-free supernatants from mixed cultures of *P. chrysogenum *and *B. subtilis*.** Mutanase activity was measured after addition of supernatant samples of mixed cultures of *P. chrysogenum* and *B. subtilis* grown in shake flasks (○), of a 24-h mixed chemostat culture (●) to a 24-h batch culture of *P. chrysogenum* and in culture grown in presence of 0.1% of mutan (α 1,3 glucan) (▲), of 0.1% of pullulan (α-1,4-α-1,6-glucan), of 0.1% of dextran (α1,6 glucan) (×) and 0.1% of β1,3 glucan (∆) The presented data are averages and standard deviations of triplicate experiments. We It is worth mentioning that the residual mutanase activity (0.020 U.ml^-1^) observed at the start of the *P. chrysogenum* shake flask cultures spiked with supernatant of mixed culture originated from the inoculum. Data are presented as average ± mean deviation of three independent experiments.

The upregulation of mutanase genes in *P. chrysogenum* could theoretically be induced by compounds released by *B. subtilis*. To test whether extracellular bacterial signaling molecules were sufficient to triggering fungal mutanase synthesis, 24 h-old *P. chrysogenum* shake-flask cultures cultures were aseptically supplied with either 30 ml of filter-sterilized supernatant of a pure *B. subtilis* culture, 30 ml of filter-sterilized supernatant a from mixed chemostat culture or with heat-inactivated *B. subtilis* cells. These cultures were allowed to grow for another 72 h and samples were taken after 0 h, 24 h, 48 h and 72 h for determination of mutanase activity (Figure 6). Mutanase production was not observed when *P. chrysogenum* was co-cultured with *B. subtilis* supernatant or heat inactivated *B. subtilis* cells. However, the *P. chrysogenum* cultures did produce mutanase when inoculated with filter sterilized supernatants from 24-h mixed cultures of *P. chrysogenum* and *B. subtilis*. Mutanase activity was also observed in control shake flask mixed culture of *P. chrysogenum* and *B. subtilis* (Figure 6). In any case the growth of *P. chrysogenum* was not inhibited by the *B.subtilis* culture fractions. In addition, mutan (α 1,3 glucan) was sufficient to induce mutanase production in *P. chrysogenum* supernatant. In contrast addition to the culture medium of pullulan (α1,4-α1,6 glucan), dextran (α1,6 glucan) and β1,3 glucan did not lead to mutanase induction (Figure 6).

### Cloning and functional characterization of two putative *P. chrysogenum* mutanase genes

To functionally characterize the two upregulated *P. chrysogenum* putative mutanase genes, the coding regions of Pc12g07500 and Pc12g13330 were expressed in *S. cerevisiae*, a eukaryotic microorganism that does not itself express mutanase. The predicted coding sequence of the putative mutanase Pc12g07500 included a 63-bp 5′ sequence predicted to encode an N-terminal signal peptide. Therefore, Pc12g07500 cDNA including the signal peptide was PCR amplified from mRNA isolated from *P. chrysogenum* mycelium, pooled from different 72-h co-cultures with *B. subtilis.* The cDNA was inserted in plasmid pAG426GPD-ccdB [43], which placed it under control of the constitutive *TDH3* promoter and *CYC1* terminator (pUDE230). When grown in shake-flask cultures on a synthetic medium containing 20 g l^-1^ glucose, *S. cerevisiae* strain IME208, carrying this plasmid, showed a specific growth rate of 0.29 ± 0.02 h^-1^. The sequence of the gene in the entry clone was confirmed by restriction analysis and DNA sequencing. Presence of the expression plasmid was confirmed by diagnostic PCR, and transcription of Pc12g07500 in *S. cerevisiae* IME208 confirmed by RT-qPCR (Table 2). However, mutanase production was neither observed in cultures of this strain nor in cultures of a negative control strain (*S. cerevisiae* CEN.PK113-5D containing the control plasmid p426GPD [44]).

**Table 2:** **Transcript levels of heterologously expressed putative mutanase genes from *P. chrysogenum *** **in *S. cerevisiae***

**Gene and *S. cerevisiae *** **strain/relevant genotype**	**Gene expression relative to reference gene *ACT1***
Pc12g07500 IME208 [pUDE 230 (*TDH3*_*p*_*::Pc12g07500::*CYC1_t_)]	1.52 ± 0.16
Pc12g07500 IME209 [pUDE 249 (*TDH3*_*p*_*::MF(Alpha)1*^*1–270*^*-Pc12g07500-*∆^1–63^*::CYC1*_*t*_)]	1.49 ± 0.33
Pc12g13330 IME210 [pUDE 250 (*URA3 TDH3*_*p*_*::MF(Alpha)1*^*1–270*^*-Pc12g13330::CYC1*_*t*_)]	1.13 ± 0.08

Expression levels were estimated by RT-qPCR using *ACT1.* Data are average ± mean deviation of duplicate analyses.

The absence of mutanase activity in culture supernatants of *S. cerevisiae* IME208 might originate from a lack of recognition of the *Penicillium* signal peptide by the yeast secretion machinery. To circumvent this potential problem, the first 63 nucleotides of Pc12g07500 were replaced by the signal peptide of the *S.cerevisiae* alpha mating factor [45]. The *S. cerevisiae* strain IME209, expressing the α-factor pre-pro-peptide – P12g07500 chimera, showed a strongly impaired specific growth rate (0.09 ± 0.01 h^-1^) on glucose synthetic medium. A mutanase activity of 0.03 U.ml^-1^ was detected in culture supernatants, confirming that Pc12g07500 encodes an active mutanase.

The amino acid sequence analysis of the second putative mutanase gene, Pc12g1330, did not reveal the presence of a signal peptide. Therefore, its cDNA was directly cloned behind the α-factor pre-pro-peptide sequence to promote secretion of the enzyme by *S. cerevisiae*. In shake flask cultures of the resulting *S. cerevisiae* strain IME210 on glucose, it showed a specific growth rate of 0.27 ± 0.01 h^-1^. No mutanase activity was detected in IME210 culture supernatant, despite confirmation by RT-PCR that the gene fusion was transcribed (Table 2).

## Discussion

### General responses of *P. chrysogenum* to co-cultivation with *B. subtilis*

While co-cultivation experiments under laboratory conditions cannot fully capture the entire complexity of microbial interactions in natural environment, they do enable identification of specific mechanisms by which micro-organisms interact [18,20-22]. The present study focused on responses of *P. chrysogenum* to co-cultivation with the *B. subtilis*. A substantial part of the transcriptional response of *P. chrysogenum* to co-cultivation could be attributed to competition for glucose, the growth-limiting nutrient used for the chemostat studies. However this has not been experimentally tested since our fermenters were not equipped with rapid sampling port and the actual supernatant sampling procedure was far too long to trust the measured values. When *B. subtilis* was inoculated in a glucose-limited chemostat of *P. chrysogenum*, the bacterium was able to grow faster than the fungus and, over a 72 h period, was well on the way to outcompete it highlighting a better affinity (μ_max_/Ks) of the bacterium for the limited nutrient. The strong up-regulation of *P. chrysogenum* genes including sugar transporters (Figure 3, Cluster 4), including genes encoding putative high-affinity transporters, represents a typical response of a micro-organism that is confronted with competition for glucose as the growth-limiting nutrient [46].

The gene Pc12g10440, which showed the strongest upregulation during co-cultivation with *B. subtilis*, shares strong similarity with the alternative oxidase gene *aox1* from *A. niger*. Alternative oxidases in fungi and plants represent bypass energy coupling sites in the mitochondrial respiratory chain. Their expression is upregulated under a variety of stress conditions, including changes in the supply of, or demand for, carbon, reducing power, and ATP [47]. Interestingly, alternative oxidases have also been directly implicated in the defense against microbes and, in the case of pathogens, with host cells. For example, expression of alternative oxidase in *Nicotania attenuata* has been shown to contribute to resistance against pathogenic *Pseudomonas syringae*[48] and, in pathogenic fungi, the enzyme counteracts the host’s immune system [49-51].

The side-chain precursor phenylacetic acid, whose addition is essential for high-level production of penicillin G by *P. chrysogenum*, was not included in the growth media used for the present study. *P. chrysogenum* is able to synthesize phenylacetic acid by itself, using L-phenylalanine as a precursor [10]. However, co-cultivation did not affect expression of genes involved in phenylalanine production or catabolism. This result indicated that, at least in this strain background, co-cultivation of a penicillin-sensitive Gram positive bacterium, did not trigger penicillin production. Although the Wisconsin strain of *P. chrysogenum* represents an early strain in the penicillin strain improvement lineage [31], we cannot exclude the possibility that its penicillin biosynthetic pathways had already been deregulated as a result of strain improvement, thereby masking induction by the presence of bacterial cells. However, unexpectedly and in contrast to a previous study on co-cultivation of *Aspergillus nidulans* with *Streptomyces hygroscopicus*[18], *P. chrysogenum* did not induce other secondary metabolite biosynthetic gene clusters upon in response to the presence of *B. subtilis* (Figure 4).

### Expression of fungal mutanase activity results from cross talk between *P. chrysogenum and B. subtilis*

Although we were unable to connect co-cultivation of *P. chrysogenum* with a prokaryote to production of low-molecular weight fungal metabolites, co-cultivation clearly triggered the expression of two genes encoding putative α-1, 3 glucanases (Pc12g07500 and Pc12g13330). Synthesis of mutanase (α-1,3-glucanase, EC 3.2.1.59) is induced in several fungi and bacteria when mutan (α-1,3-glucan) or oligosaccharides such as raffinose are present in the growth medium [52-54]. Mutanase production by *Trichoderma harzianum* is triggered by sugars or molecule with α-1,3 linkages, consistent with the ability of the enzyme to act on α-1,3 linkages in bacterial biofilms and extracellular polysaccharides such as α-mutan [55].

Upregulation of the putative mutanase genes Pc12g07500 and Pc12g13330 required the presence of the two microorganisms simultaneously. Supernatant of pure *B. subtilis* culture or heat inactivated *B. subtilis* cells were not sufficient to induce the expression of these genes (Table 2), suggesting that the triggering molecule(s) is (are) produced by *B. subtilis* in response to the presence of *P. chrysogenum.* Induction of the mutanase genes during co-cultivation could, for example, result from the formation of α-1,3 glucan containing exopolysaccharide by *B. subtilis* in response to co-cultivation by the fungus. This bacterial response could then be countered by the production of mutanase by the fungus. Several pathogenic bacteria in environment reside in exopolysaccharide biofilm which protects bacterial cells from antibiotics [56]. If enzymes produced against these pathogenic bacterial exopolysaccharides in mixed culture enable bacterial biofilm degradation, this might potentiate fungal antibiotics by facilitating access to the bacteria.

### *P. chrysogenum* Pc12g07500 is an atypical fungal mutanase without a mutan binding domain

**Figure 7:**
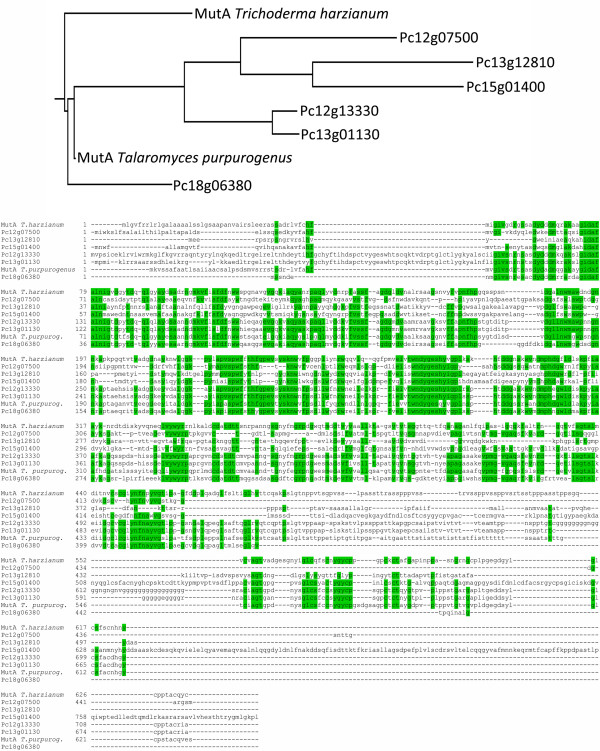
**Comparison of putative mutanase (α-1,3 glucanase) encoding genes in *P. chrysogenum.*** Top panel- Phylogenetic tree based on Clustal-X alignment of predicted amino acid sequences of *P. chrysogenum* Pc12g07500 (accession number: CAP80377.1), Pc12g13330 (CAP80960), Pc13g12810 (CAP92350), Pc13g01130 (CAP91182), Pc15g01400 (CAP83026), Pc18g06380 (CAP94862), *Trichoderma harzianum* MutA (ADZ45396.1) and *Talaromyces purpurogenus* MutA (AF214481_1). The tree was constructed using TREECON for Windows. Bottom panel- Amino acid sequence alignment of the previously mentioned proteins. Amino acids highlighted in green represent positions that are conserved in at least 50% of the aligned sequences.

Of the two putative mutanase genes that were upregulated during co-cultivation, only Pc12g07500 yielded extracellular mutanase activity when expressed in *Saccharomyces cerevisiae*. The genome sequence of *P. chrysogenum*[2] harbors six genes that share similarity to the *mutA* genes from *Trichoderma harzianum* and *Talaromyces purpurogerus* (formely known as *Penicillium purpurogenum*) (Figure 7). Comparison of the predicted amino acid sequences of these six putative mutanases revealed that only two of them (Pc12g07500 and Pc15g01400) were predicted to contain a signal peptide. Interestingly, the amino acid sequence analysis also revealed that, in contrast to previously characterized fungal mutanases, the Pc12g07500 amino acid sequence was much shorter and missed the C-terminal sequence that corresponds to the α-mutan binding domain (Figure 7).

Pc12g13330, which showed a higher sequence similarity to *T. purpurogenus MutA*, did not harbor an N terminal sequence corresponding to a signal peptide, but did contain a C-terminal extension corresponding to the mutan binding motif. Of the six *P. chrysogenum* mutanases, not only Pc12g07500, but also Pc18g06380 missed the α-mutan binding domain. However, in contrast to Pc12g07500, Pc18g06380 did not appear to carry a signal peptide and was not expressed during co-cultivation with *B. subtilis*.

Hitherto, fungal mutanases have been described as strictly extracellular enzymes [42,57]. Interestingly, several of the genes with strong sequence similarity to mutanase genes in the *P. chrysogenum* genome appear to lack excretion signals. 1, 3-α-D-glucan has been detected in cell walls of *Aspergilllus wentii*[58] and *Schizosaccharomyces pombe*[59], intracellular mutanase-like enzymes may play a role in processing and/or degradation of fungal cell wall components. Expression of the *P. chrysogenum* mutanase gene Pc12g07500 in *S. cerevisiae* (strain IME209) had a strong negative effect on yeast growth. This is an intriguing observation, since 1,3-α-D-glucan has not been reported to occur in *S. cerevisiae* cell walls. Detailed analysis of the mode of growth inhibition of mutanase-expressing strains may yield new insights in yeast cell wall composition and biosynthesis.

## Conclusion

The present study demonstrates that co-cultivation experiments with defined mixed cultures provide a useful tool for identifying microbial interactions. Under the experimental conditions, *B. subtilis* successfully competed with *P. chrysogenum* for the glucose, the growth-limiting nutrient. The fungus did not respond to this competition by induction of pathways for antimicrobial secondary metabolites. However, a co-cultivation-dependent expression of an extracellular fungal mutanase was observed, which was triggered by (an) extracellular compound(s) produced by the mixed cultures but not by pure cultures of the two organisms. Heterologous expression in *S. cerevisiae* revealed that of the *P. chrysogenum* genes induced during co-cultivation with *B. subtilis* encoded an extracellular mutanase that differed from previously described fungal mutanases due to the absence of an α-mutan binding domain.

## Methods

### Strains

*P. chrysogenum* Wisconsin 54–1255 (ATCC 28089) was obtained from DSM-Anti-Infectives (Delft, The Netherlands) as spores on rice grains. *B. subtilis* NCCB 70064, obtained from The Netherlands Culture Collection of Bacteria (NCCB) (http://www.cbs.knaw.nl/collections), was grown at 30°C in shake flasks containing 100 ml of synthetic medium (see below). 30% (v/v) glycerol was added to a 24-h old culture and 1 ml aliquots were stored at -80°C. *S. cerevisiae* strains used and constructed in this study (Table 3) are members of the CEN.PK strain family [60,61]. Stock cultures of all *S. cerevisiae* strains were grown in shake flasks containing 100 ml of synthetic medium (see below) with 20 g l^-1^ of glucose as the carbon source. When mid-exponential phase was reached, sterile glycerol was added to make 30% glycerol (v/v) and 1 ml aliquots were stored at -80°C.

**Table 3:** ***S. cerevisiae *** **strains used and constructed in this study**

**Strain**	**Genotype**	**Reference**
CEN.PK 113-5D	*MATa SUC2 MAL2-8C ura3-52*	Euroscarf
CEN.PK 113-7D	*MATa SUC2 MAL2-8C*	Euroscarf
IME 208	*MATa SUC2 MAL2-8C ura3-52* pUDE 230 (*URA3 TDH3*_*p*_*::Pc12g07500::CYC1*_*t*_)	This work
IME 209	*MATa SUC2 MAL2-8C ura3-52* pUDE 249 (*URA3 TDH3*_*p*_*::MF(Alpha)1*^*1–270*^*-Pc12g07500-*∆^1–63^*:: CYC1_*t*_)	This work
IME 210	*MATa SUC2 MAL2-8C ura3-52* pUDE 250 (*URA3 TDH3*_*p*_*::MF(Alpha)1*^*1–270*^*-Pc12g13330:: CYC1*_*t*_)	This work
IME 211	*MATa SUC2 MAL2-8C ura3-52* p426GPD (*URA3 TDH3*_*p*_*::-:: CYC1*_*t*_)	This work

*Deletion of bases from gene.

### Medium composition and preparations

Synthetic medium for growth of *P. chrysogenum* was prepared as described [62] and contained, per liter of demineralized water, 8.25 g glucose monohydrate, 0.8 g KH_2_PO_4_, 3.5 g (NH_4_)_2_SO_4_, 0.5 g MgSO_4_.7H_2_O and 10 ml trace elements solution. The pH was set to 5.5 by addition of 2 M KOH. The trace element solution contained 15 g l^-1^ Na_2_EDTA.2H_2_O, 0.5 g l^-1^ Cu_2_SO_4_.5H_2_O, 2 g l^-1^ MnSO_4._H_2_O, 2 g l^-1^ ZnSO_4_.7H_2_O, 4 g l^-1^ FeSO_4_.7H_2_O and 0.5 g l^-1^ CaCl_2_.2H_2_O. The pH of the trace element solution was set at 6.0 by the addition of NaOH. Concentrated medium without glucose was heat autoclaved at 121°C. After media sterilization, a concentrated glucose solution, separately autoclaved at 110°C, was added. For cultivation of *B. subtilis*, the same medium was used, but with the addition of 50 mM MES and with an initial pH of 6.5. Synthetic medium for growth of *S. cerevisiae* contained, per liter of demineralized water, 5 g (NH_4_)_2_SO_4_, 3 g KH_2_PO_4_, 0.5 g MgSO_4_.7·H_2_O and trace elements [5]. Vitamins [5] were added after heat sterilization of the medium at 120°C for 20 min. Glucose was separately sterilized at 110°C and added to a final concentration of 20 g L^-1^.

### Pure and mixed-culture growth experiments

*P. chrysogenum* Wisconsin 54–1255 was grown in steady-state, aerobic and glucose-limited chemostat cultures. Chemostat cultures were grown at 25°C in 2 l turbine-stirred bioreactors (Applikon, Schiedam, The Netherlands) with a working volume of 1.8 L, as described previously [5,6,62]. In the batch phase a working volume of 1.5 L was maintained. The pH was adjusted to 6.5 and then kept constant at 6.5 through the automatic addition of 2 M NaOH. The bioreactor was sparged with air at a flow rate of 900 ml/min using a Brooks mass-flow controller (Brooks Instruments, Hatfield, PA). Spores immobilized on rice grains were welled in 100 ml sterilized water until the water became dark green. The liquid then was transferred to a new flask and served as inoculum. To protect the germination process the stirrer speed was initially set at 350 rpm. The dissolved-oxygen concentration was continuously monitored with an oxygen electrode (AppliSens, Schiedam, The Netherlands). When dissolved oxygen reached 60% of air saturation, the stirrer speed was increased to 500 rpm and, subsequently, to 750 rpm to keep dissolved oxygen concentration above 50% of air saturation. To prevent excessive foaming, antifoam Silcolapse 5020 (Bluestar Silicones, East Brunswick NJ) was discontinuously added at timed intervals (6 s on, every 7 min, 1.0 ml h^-1^). The off-gas was cooled using a condenser (4°C) and dried with a Perma Pure dryer (MD-110- 48P-4, Permapure, Toms River, NJ). Concentrations of CO_2_ and O_2_ in the off-gas were measured with a NGA 2000 Rosemount analyzer (Rosemount Analytical, Solon, OH). Off-gas flow rate was measured with a SAGA digital flow meter (Ion Science, Cambridge, United Kingdom). The continuous-feed phase was initiated when the CO_2_ concentration in the off gas reached 0.3%. A dilution rate of 0.03 h^-1^ was set by continuous addition of fresh medium with a calibrated peristaltic pump (Masterflex, Vernon Hills, IL). To keep the culture volume constant, culture broth was automatically removed at regular time intervals by applying an overpressure to the reactor by a system of valves [63].

Precultures of *B. subtillis* were grown by inoculating 1 ml of *B. subtilis* glycerol stock into 100 ml of glucose synthetic medium. After incubation in an orbital shaker at 30°C and at 200 rpm for 24 h, a volume of 10 ml was inoculated into 200 ml of glucose synthetic medium in a 1-l shake flask and incubated at 30°C (200 rpm). After 24 h of incubation, when the culture had reached exponential phase, cells were separated aseptically by centrifuging for 20 min at 4°C and at 13000 × *g*, washed twice and re-suspended in 50 ml sterile *P. chrysogenum* synthetic medium. The resulting cell suspension, containing ca. 1.8 g dry weight *B. subtilis* biomass was then aseptically added to to a steady-state chemostat culture of *P. chrysogenum* bioreactor. The resulting mixed culture was then followed for 72 h while samples were taken for analysis of biomass and metabolites, microscopic observations and transcriptome analysis (samples times 5 h, 24 h, 48 h and 72 h after addition of *B. subtilis*).

Effects of *B. subtilis* culture supernatant, supernatant from mixed cultures, and heat-inactivated *B. subtilis* cells and addition of 0.1% mutan (kindly provided by Prof. Malgorzata Pleszczynska, Maria Curie-Sklodowska University, Lublin, Poland), 0.1% pullulan (Megazyme International Wicklow, Ireland) and 0.1% dextran (Megazyme) on *P. chrysogenum* were checked in shake-flask experiments. *P. chrysogenum* was grown in 100 ml glucose synthetic medium in 500 ml flask at 25°C, shaken at 200 rpm. Fermentation broth of exponentially growing *B. subtilis* cultures and culture samples taken from 24 h mixed chemostat cultures were filtered through 0.20 μm filters (Supor-200, 47 mm, Pall Life Sciences, East Hills, NY). 30 ml *B. subtilis* supernatant and 30 ml of mixed-culture supernatant were added aseptically to different 24-h *P. chrysogenum* cultures. Effects of addition of mutan, pullulan and dextran on mutanase induction in *P. chrysogenum* were checked in shake-flask experiments. The strain was grown in synthetic medium with 0.1% of mutan, 0.1% pullulan or 0.1% dextran. These cultures were allowed to grow for another 72 h, during which samples were taken at 24 h intervals for determining mutanase activity. All cultivation experiments were performed in triplicate.

Heat-inactivated *B. subtilis* cells were generated by autoclaving 30 ml of exponentially growing *B. subtilis* culture for 20 min at 121°C. Killed cells were separated aseptically by centrifuging for 20 min at 4°C and at 13000 × *g*, washed twice and re-suspended in 10 ml of with sterile synthetic medium. The resulting suspension was then aseptically added to a 24-h *P. chrysogenum* shake-flask culture. Control experiments were performed by using exponentially growing *B. subtilis* cells instead of killed cells.

### Analytical methods

#### ***Determination of culture dry weight***

For *P. chrysogenum*, 10 ml fermentation broth was filtered over preweighed glass fiber filters Type A/E (Pall Life Sciences). The filters were washed with demineralised water and dried for 20 min at 600 W in a microwave oven and were weighed immediately. For *B. subtilis*, 10 ml fermentation broth was filtered over preweighed 0.45 μm filters (Supor-450, 47 mm, Pall Life Sciences). The filters were washed with demineralised water and dried for 20 min at 350 W in a microwave oven and were weighed immediately. For mixed cultures, 10 ml of culture sample was filtered over preweighed glass fiber filters. The filters were washed three times with 10 ml of demineralised water so that all the bacterial biomass passed through the glass fiber filters; the combined filtrate was collected. The glass fiber filters were dried for 20 min at 600 W in a microwave oven and were weighed immediately. The collected filtrate was passed through preweighed 0.45 μm filters. The filters were washed with demineralised water and dried for 20 min at 350 W in a microwave oven and were weighed immediately.

#### ***Glucose measurement***

Glucose in culture supernatants was analyzed on a Waters 2695 HPLC (Waters, Milford, MA) equipped with an Aminex HPX-87H column (Bio-rad, Hercules, CA) which was coupled to a Waters 2487 dual λ absorbance detector (Waters) at 60°C with 5 mM H_2_SO_4_ as the mobile phase. Supernatant was separated from the biomass by filtration using a 0.2 μm filter.

#### ***Microscopy***

Microscopic observations of the mixed culture were made with a Zeiss Axio imager D1 equipped with an Axio Camera (Carl Zeiss, Jena, Germany).

#### ***Mutanase assay***

Mutanase activity was assayed by incubating 0.5 ml of culture supernatant and 0.5 ml of a 0.2% (w/v) suspension of dextranase-treated mutan in 0.05 M potassium phosphate buffer, pH 6.5 at 45°C (kindly provided by Prof. Malgorzata Pleszczynska, Maria Curie-Sklodowska University, Lublin, Poland). After 60-min incubation with shaking, the insoluble mutan was centrifuged (13000 rpm for 5 min) and the released reducing sugars were estimated by the Somogyi-Nelson method [64] using D-glucose as a standard. One unit of mutanase activity (U) was defined as the amount of the enzyme that catalyzes the release of reducing sugars equivalent to 1 μmol per minute under the experimental conditions [55].

### RNA extraction

To isolate *P. chrysogenum* RNA, mixed-culture samples were filtered through glass fiber filters within seconds, the filter with mycelium was quenched in liquid nitrogen and stored at -80°C until use. Total RNA was extracted by using Trizol reagent and acid-phenol chloroform for RNA extraction and purified by the RNeasy mini kit by Quiagen (Venlo, Netherlands). Quality of the purified RNA was verified by the Agilent 2100 bioanalyzer (Agilent, Santa Clara, CA). To isolate *S. cerevisiae* RNA, cells were collected by rapid filtration and stored at -80°C until use. Total RNA extraction was performed using acid-phenol chloroform for RNA extraction and 3 M Na acetate as described previously in [65].

### Microarray and transcriptome analysis

Chemostat culture samples (60 ml) were rapidly sampled and filtered over a glass fiber filter (Type A/E, Pall Life Sciences, East Hills, NY). The filter containing the mycelium was immediately wrapped in aluminum foil, quenched in liquid nitrogen and stored at -80°C. Samples were processed as described [5,6,8,10]. Acquisition and quantification of microarray images and data filtering were performed using Affymetrix GeneChip Operating Software (GCOS version 1.2). Arrays were globally scaled to a target value of 100, using the average signal from all probe sets. Arrays were analyzed as previously described [10]. Significant changes in expression of the replicate arrays experiments were assessed statistically by using the software Significance Analysis of Microarray (SAM version 1.21) [33]. Fold change (FC) was set to 2 and false discovery rate to 2%. Transcriptome data analyzed in this study have been deposited at the Genome Expression Omnibus database http://www.ncbi.nlm.nih.gov/geo/) under the accession number GSE53286. Expression data were K-mean clustered using GeneData Expressionist software version 5.0 (Genedata, Basel, Switzerland). Clusters were analyzed for enrichment of MIPS categories, assessed by Fisher’s Exact test employing hypergeometric distribution with a p-value cut-off of 5.10^-4^ (with Bonferroni correction) [66].

### Cloning and functional characterization of *P. chrysogenum* genes in *S. cerevisiae*

Pc12g07500 cDNA was PCR amplified using Phusion Hot-Start Polymerase (Finnzymes, Landsmeer, The Netherlands), using primer pairs AttbPc12g07500-FW/AttbPc12g07500-RV (Table 4) and *P. chrysogenum* cDNA prepared from mRNA of pooled mixed-culture samples. The purified PCR product was cloned in pDONR™ 221 (Invitrogen, Carlsbad, CA) by the BP clonase™ reaction, resulting in entry clone pUD254. This entry clone was then further cloned, using the LR clonase™ reaction, into the destination *S. cerevisiae* expression vector pAG426GPD-*ccdb*[43], which carries a *TDH3* promoter and *CYC1* terminator upstream and downstream of the att recombination sites. This resulted in the expression vector pUDE230, which carries Pc12g07500 cDNA under the control of the *TDH3* promoter.

**Table 4:** Oligonucleotide primers used in this study

**Primer name**	**Sequence**
Cloning	
AttbPc12g07500-RV	GGGGACAAGTTTGTACAAAAAAGCAGGCTATGATTTGGAAATCTCTCTTTAGTGCTTTGGCC
AttbPc12g07500-RV	GGGGACCACTTTGTACAAGAAAGCTGGGTTCACATGCTACCTCTAGCC
AttbMF(Alpha)1-FW	GGGGACAAGTTTGTACAAAAAAGCAGGCTATGAGATTTCCTTCAATTTTTACTGC
MF(Alpha)1 *Spe*1-RV	GGCTAGCACTAGTGTAAGCTTCAGCCTCTCTTTTCTCG
Pc12g07500 *Spe*1-FW	CGTTAGACTAGTGCCCCAGCTCTCGATTCAGAGC
Expression (RT-qPCR)	
Pc12g07500 208 FW	TCCGGGTACCTTGACTGTGC
Pc12g07500 208 RW	CCCGAGCCACCTTGAAAGAC
Pc12g07500 209 FW	TCCGGGTACCTTGACTGTGC
Pc12g07500 209 RV	CCCGAGCCACCTTGAAAGAC
Pc12g13330 210 FW	GACGCTTTGCAACCAGAAGG
Pc12g13330 210 RV	TGGGACGGTACCCAAAGATG
3′ FW ACT1	GGCTTCTTTGACTACCTTCCA
3′ RV ACT1	AGAAACACTTGTGGTGAACGA

To facilitate secretion of the mature protein in *S. cerevisiae,* the signal peptide region in Pc12g07500 was replaced with *S. cerevisiae* α-factor prepropeptide [45]. The gene fragment coding for Pc12g07500 without signal peptide was PCR amplified using primer pair Pc12g07500 SpeI-FW/Attb Pc12g07500-RV (Table 4) and *P. chrysogenum* cDNA pool prepared from mRNA isolated from mixed culture sample. *S. cerevisiae* α-factor prepropeptide cDNA was PCR amplified using primer pair Attb MF(Alpha)1-FW/MF(Alpha)1 SpeI-RV (Table 4) and DNA sample isolated from *S. cerevisiae* CEN.PK113-7D [67]. Both the PCR products were purified and restricted with SpeI, followed by ligation with T4 DNA ligase. The ligation mixture was used as template to perform PCR using primer pair Attb MF(Alpha)1-FW/Attb Pc12g07500-RV and the target DNA fragment was size-selected and gel purified [68]. The purified PCR product was cloned in pDONR™ 221 in a BP clonase™ reaction to get entry clone pUD288. The obtained entry clone pUD 288 was further recombined in LR clonase™ reaction with the destination plasmid pAG426GPD-ccdb [43] to get the expression clone pUDE249.

A synthetic gene containing *S. cerevisiae* α-factor prepropeptide and HA tag with Pc12g13330 (2474 bp) was gene optimized using JCat [69] and procured from GenScript (Piscataway, NJ). Restriction sites SpeI (upstream) and XhoI (downstream) were introduced in the synthetic gene. The synthetic gene and p426GPD [44] were restricted with SpeI and XhoI and fragments were recovered from agarose gels with a commercial kit (Zymoclean, Zymo Research). The synthetic gene was ligated into SpeI/XhoI digested p426GPD, resulting in plasmid pUDE250. The expression vector pUDE250 carries Pc12g13330 cDNA placed under the control of the *TDH3* promoter.

Transformations of recombination reaction products into competent *E. coli* JM109 were performed with the Z-Competent *E. coli* transformation kit (Zymo Research, Orange, CA) and plated on LB media containing either ampicillin (100 mg l^-1^) or kanamycin (50 mg l^-1^). The gene sequences in entry clone pUD254 and pUD288 were checked by restriction digestion and DNA sequencing performed at BaseClear, BV (Leiden, The Netherlands).

Plasmids pUDE230, pUDE249, pUDE250 and the *URA3*-bearing ‘empty vector’ plasmid p426GPD [44] were transformed into *S. cerevisiae* CEN.PK113-5D using the lithium acetate protocol [70]. After transformations with the yeast expression plasmid, cells were plated on chemically defined medium. Successful insertion of multicopy plasmids was verified by diagnostic PCR using the primer pairs used for PCR amplification. *S. cerevisiae* CEN.PK113-5D containing plasmids pUDE230, pUDE249, pUDE250 and p426GPD were designated as IME208, IME209, IME210 and IME211 respectively (Table 3).

### Quantitative real time PCR (RT-qPCR)

For preparation of cDNA for RT-qPCR, synthesis of first-stand cDNA was carried out using a QuantiTect reverse transcription kit (Qiagen, Venlo, Netherlands). Total RNA was isolated from shake flasks of *S. cerevisiae* strains IME208, IME 209 and IME 210 according to the manufacturer’s recommendations. Primers for real-time PCR (Table 4) were designed with Clone Manager 9 (Scientific & Educational Software, Cary, NC). Basic Local Alignment Search Tool (BLAST) [71] analysis was used to verify that each primer pair was specific for a particular *P. chrysogenum* gene, and would not cross-react with the *S. cerevisiae* genome. RT-qPCR assays were carried out using SYBR^®^ Green Jumpstart™ Taq ReadyMix™ (Sigma-Aldrich, St. Louis, MO) in the DNA Engine Opticon™ I system from MJ research™ according to the manufacturer’s instructions. PCR reactions on cDNA template were carried out in triplicate. The results for each growth condition were derived from two independently cultured replicates. Data were analyzed using the Opticon Monitor™ software package version 1.04, calculating Ct values. Melting curves were used to monitor the specificity of the reaction. β-Actin (*ACT1*) was used as an internal control for sample normalization. PCR reactions on each sample were conducted in triplicate. Data were analyzed by the comparative threshold cycle method [72].

## Competing interests

The authors declare that they have no competing interests.

## Authors’ contributions

JTP and J-MD designed the study. IB, TV, DvD performed the experiments. IB, TV, DvD, JTP and J-M Daran performed the data analysis and prepared the manuscript. All authors read and approved the final manuscript.

## Supplementary Material

Additional file 1: Table S1 K-mean clustered gene expression profiles over the course of co-cultivation of *P. chrysogenum* with *B. subtilis*. The numerical data represent the average of the normalized hybridization data. Click here for file
